# Supplementary material for: End Sequence Analysis Toolkit (ESAT) expands the extractable information from single-cell RNA-seq data
Source: Genome Res. 2016 Oct;26(10):1397–410. doi: 10.1101/gr.207902.116 (PMC5052061; doi:10.1101/gr.207902.116)
Supplement: Supplemental Material [file supp_gr.207902.116_Supp_Materials.tar.gz › ESAT-esat_v0.1/doc/html/classbroad_1_1pda_1_1rnai_1_1_extract_sequence.html]

ESAT: broad.pda.rnai.ExtractSequence Class Reference


ESAT


- Main Page
- Related Pages
- Packages
- Classes
- Files

- Class List
- Class Index
- Class Hierarchy
- Class Members

All Classes Namespaces Files Functions Variables Enumerator Pages

- broad
- pda
- rnai
- ExtractSequence

Static Public Member Functions |
List of all members

broad.pda.rnai.ExtractSequence Class Reference

|  |  |
| --- | --- |
| Static Public Member Functions | |
| static Map | getSequenceForGene (Collection< Gene > alignments, String genomeDirectory, boolean repeatMask, Map< String, IntervalTree< Alignments >> okRepeats) throws Exception |
|  | |
| static Sequence | getFirstSequence (String fastaFile) throws IOException |
|  | |
| static Map | splitByChr (Collection< Gene > genes) |
|  | |
| static String | getSequenceForGene (Gene gene, Sequence chrom, boolean repeatMask, Map< String, IntervalTree< Alignments >> okRepeats, boolean stranded) throws Exception |
|  | |
| static String | getSequenceForGene (Gene gene, Sequence chrom, boolean repeatMask, Map< String, IntervalTree< Alignments >> okRepeats) throws Exception |
|  | |
| static String | getSequenceUnoriented (Alignments align, Sequence chrom, boolean repeatMask, Map< String, IntervalTree< Alignments >> okRepeats) throws Exception |
|  | |
| static String | getSequenceUnoriented (Alignments align, Sequence chrom, boolean repeatMask) throws Exception |
|  | |
| static String | getSequenceForGene (Gene gene, String genomeDirectory, boolean repeatMask) throws Exception |
|  | |
| static String | getGenomeSequence (GenomicAnnotation annot, String genomeDirectory, boolean repeatMask) throws Exception |
|  | |
| static String | getSequenceUnoriented (Alignments align, String genomeDirectory, boolean repeatMask, Map< String, IntervalTree< Alignments >> okRepeats) throws Exception |
|  | |
| static String | reverseComplement (String seq) |
|  | |
| static void | writeFasta (String save, Map< String, Collection< Gene >> genesByChr, String genomeDir, boolean repeatMask) throws Exception |
|  | |
| static void | main (String[] args) throws Exception |
|  | |

## Member Function Documentation

|  |  |  |  |  |  |
| --- | --- | --- | --- | --- | --- |
| static Sequence broad.pda.rnai.ExtractSequence.getFirstSequence | ( | String | *fastaFile* | ) | throws IOException |

static

Here is the caller graph for this function:

|  |  |  |  |
| --- | --- | --- | --- |
| static String broad.pda.rnai.ExtractSequence.getGenomeSequence | ( | GenomicAnnotation | *annot*, |
|  |  | String | *genomeDirectory*, |
|  |  | boolean | *repeatMask* |
|  | ) |  | throws Exception |

static

Suggestion: Use classes in net.sf.picard.reference instead, which are WAY faster because they use an indexed FASTA file

Parameters
:   |  |  |
    | --- | --- |
    | annot |  |
    | genomeDirectory |  |
    | repeatMask |  |

Returns

Exceptions
:   |  |  |
    | --- | --- |
    | Exception |  |

Here is the call graph for this function:

|  |  |  |  |
| --- | --- | --- | --- |
| static Map broad.pda.rnai.ExtractSequence.getSequenceForGene | ( | Collection< Gene > | *alignments*, |
|  |  | String | *genomeDirectory*, |
|  |  | boolean | *repeatMask*, |
|  |  | Map< String, IntervalTree< Alignments >> | *okRepeats* |
|  | ) |  | throws Exception |

static

Here is the call graph for this function:

Here is the caller graph for this function:

|  |  |  |  |
| --- | --- | --- | --- |
| static String broad.pda.rnai.ExtractSequence.getSequenceForGene | ( | Gene | *gene*, |
|  |  | Sequence | *chrom*, |
|  |  | boolean | *repeatMask*, |
|  |  | Map< String, IntervalTree< Alignments >> | *okRepeats*, |
|  |  | boolean | *stranded* |
|  | ) |  | throws Exception |

static

Here is the call graph for this function:

|  |  |  |  |
| --- | --- | --- | --- |
| static String broad.pda.rnai.ExtractSequence.getSequenceForGene | ( | Gene | *gene*, |
|  |  | Sequence | *chrom*, |
|  |  | boolean | *repeatMask*, |
|  |  | Map< String, IntervalTree< Alignments >> | *okRepeats* |
|  | ) |  | throws Exception |

static

Here is the call graph for this function:

|  |  |  |  |
| --- | --- | --- | --- |
| static String broad.pda.rnai.ExtractSequence.getSequenceForGene | ( | Gene | *gene*, |
|  |  | String | *genomeDirectory*, |
|  |  | boolean | *repeatMask* |
|  | ) |  | throws Exception |

static

Here is the call graph for this function:

|  |  |  |  |
| --- | --- | --- | --- |
| static String broad.pda.rnai.ExtractSequence.getSequenceUnoriented | ( | Alignments | *align*, |
|  |  | Sequence | *chrom*, |
|  |  | boolean | *repeatMask*, |
|  |  | Map< String, IntervalTree< Alignments >> | *okRepeats* |
|  | ) |  | throws Exception |

static

Here is the call graph for this function:

Here is the caller graph for this function:

|  |  |  |  |
| --- | --- | --- | --- |
| static String broad.pda.rnai.ExtractSequence.getSequenceUnoriented | ( | Alignments | *align*, |
|  |  | Sequence | *chrom*, |
|  |  | boolean | *repeatMask* |
|  | ) |  | throws Exception |

static

Here is the call graph for this function:

|  |  |  |  |
| --- | --- | --- | --- |
| static String broad.pda.rnai.ExtractSequence.getSequenceUnoriented | ( | Alignments | *align*, |
|  |  | String | *genomeDirectory*, |
|  |  | boolean | *repeatMask*, |
|  |  | Map< String, IntervalTree< Alignments >> | *okRepeats* |
|  | ) |  | throws Exception |

static

Here is the call graph for this function:

|  |  |  |  |  |  |
| --- | --- | --- | --- | --- | --- |
| static void broad.pda.rnai.ExtractSequence.main | ( | String[] | *args* | ) | throws Exception |

static

Here is the call graph for this function:

|  |  |  |  |  |  |
| --- | --- | --- | --- | --- | --- |
| static String broad.pda.rnai.ExtractSequence.reverseComplement | ( | String | *seq* | ) |  |

static

Here is the caller graph for this function:

|  |  |  |  |  |  |
| --- | --- | --- | --- | --- | --- |
| static Map broad.pda.rnai.ExtractSequence.splitByChr | ( | Collection< Gene > | *genes* | ) |  |

static

Here is the caller graph for this function:

|  |  |  |  |
| --- | --- | --- | --- |
| static void broad.pda.rnai.ExtractSequence.writeFasta | ( | String | *save*, |
|  |  | Map< String, Collection< Gene >> | *genesByChr*, |
|  |  | String | *genomeDir*, |
|  |  | boolean | *repeatMask* |
|  | ) |  | throws Exception |

static

Here is the call graph for this function:

Here is the caller graph for this function:

---

The documentation for this class was generated from the following file:

- java/broad/pda/rnai/ExtractSequence.java


---

Generated on Fri Aug 15 2014 15:11:01 for ESAT by  

 1.8.7
